# From the diagnosis of infectious keratitis to discriminating fungal subtypes; a deep learning-based study

**DOI:** 10.1038/s41598-023-49635-8

**Published:** 2023-12-14

**Authors:** Mohammad Soleimani, Kosar Esmaili, Amir Rahdar, Mehdi Aminizadeh, Kasra Cheraqpour, Seyed Ali Tabatabaei, Reza Mirshahi, Zahra Bibak-Bejandi, Seyed Farzad Mohammadi, Raghuram Koganti, Siamak Yousefi, Ali R. Djalilian

**Affiliations:** 1 Eye Research Center, Farabi Eye Hospital, Tehran University of Medical Sciences, Tehran, Iran; 2 Department of Ophthalmology and Visual Sciences, University of Illinois at Chicago, Chicago, IL USA; 3 Department of Telecommunication, Faculty of Electrical Engineering, Shahid Beheshti University, Tehran, Iran; 4 Eye Research Center, The Five Senses Health Institute, Rasoul Akram Hospital, Iran University of Medical Sciences, Tehran, Iran; 5 Translational Ophthalmology Center, Farabi Eye Hospital, Tehran University of Medical Sciences, Tehran, Iran; 6 Department of Ophthalmology, University of Tennessee Health Science Center, Memphis, USA; 7 Department of Genetics, Genomics, and Informatics, University of Tennessee Health Science Center, Memphis, USA; 8 Cornea Service, Stem Cell Therapy and Corneal Tissue Engineering Laboratory, Illinois Eye and Ear Infirmary, 1855 W. Taylor Street, M/C 648, Chicago, IL 60612 USA

**Keywords:** Corneal diseases, Diagnosis

## Abstract

Infectious keratitis (IK) is a major cause of corneal opacity. IK can be caused by a variety of microorganisms. Typically, fungal ulcers carry the worst prognosis. Fungal cases can be subdivided into filamentous and yeasts, which shows fundamental differences. Delays in diagnosis or initiation of treatment increase the risk of ocular complications. Currently, the diagnosis of IK is mainly based on slit-lamp examination and corneal scrapings. Notably, these diagnostic methods have their drawbacks, including experience-dependency, tissue damage, and time consumption. Artificial intelligence (AI) is designed to mimic and enhance human decision-making. An increasing number of studies have utilized AI in the diagnosis of IK. In this paper, we propose to use AI to diagnose IK (model 1), differentiate between bacterial keratitis and fungal keratitis (model 2), and discriminate the filamentous type from the yeast type of fungal cases (model 3). Overall, 9329 slit-lamp photographs gathered from 977 patients were enrolled in the study. The models exhibited remarkable accuracy, with model 1 achieving 99.3%, model 2 at 84%, and model 3 reaching 77.5%. In conclusion, our study offers valuable support in the early identification of potential fungal and bacterial keratitis cases and helps enable timely management.

## Introduction

Infectious keratitis (IK) is a major cause of corneal opacity, the fifth leading cause of blindness worldwide. Annual vision loss due to IK comprises approximately 2 million cases worldwide^1^. IK exerts a significant burden on the healthcare system, accounting for 1 million visits and $175 million in healthcare expenditures in the USA alone^2^. Notably, IK contributes to 10% of preventable visual impairments in the world's least-developed regions^3–5^. IK can be caused by a variety of microorganisms, including bacteria, fungi, viruses, and parasites^6^. Of these, fungal ulcers carry the worst prognosis^7^. Additionally, fungal cases can be subdivided into filamentous (e.g., *Aspergillus* spp. and *Fusarium* spp.) and yeasts (e.g., *Candida* spp.), which shows fundamental differences regarding mycological and clinical characteristics^8^.

Delays in diagnosis or initiation of appropriate treatment increase the risk of ocular complications including blindness^9^. Currently, the diagnosis of IK is mainly made based on slit-lamp examination, corneal scrapings, tissue biopsy, PCR, and confocal microscopy^10^. However, these diagnostic methods have their drawbacks, including experience-dependency, tissue damage, cost, and time consumption. Moreover, the sensitivity and accuracy of these methods are at times unsatisfying. Diagnosis of IK can be especially challenging in rural areas or in countries with limited resources due to financial constraints, the unreliability of usual laboratory diagnostic tests, and lack of access to standardized laboratory resources^11,12^. Routinely, ophthalmologists initiate empiric treatment based on clinical features, which demands extensive exposure to diverse clinical scenarios over an extended training period. Furthermore, the broad spectrum of IK presentations makes the interpretation process more complex^11,13,14^. Collectively, these issues necessitate the development of new, more accurate, and rapid diagnostic methods.

Artificial intelligence (AI), a subfield of computer science designed to mimic and enhance human decision-making, has garnered substantial attention in the medical field in recent years^15–17^. The two main subfields of AI include machine learning (ML) and deep learning (DL). Unlike ML, DL eliminates the necessity for manual feature engineering. DL uses neural networks to learn from data and perform complex tasks^18^. Previously, ocular AI research was mainly focused on diseases of the posterior segment such as diabetic retinopathy, retinopathy of prematurity, age-related macular degeneration, retinal vein occlusion, and glaucoma optic neuropathy^19–23^. However, an increasing number of studies have utilized AI in the diagnosis of anterior segment diseases such as IK^24–26^. Using AI to help diagnose and manage IK can provide a much-needed solution to the shortage of ophthalmologists and improve patient care and outcomes. AI algorithms can be trained to recognize patterns in images that are invisible to the naked eye, which allows AI algorithms to diagnose IK with exceptional accuracy. In this paper, we propose to use AI to: (1) diagnose IK (model 1 of our study), (2) differentiate between bacterial keratitis and fungal keratitis (model 2), and 3) discriminate the filamentous type from the yeast type of fungal cases (model 3) based on slit-lamp images.

## Methods

### Ethics statement

This study was in accordance with the principles of the Declaration of Helsinki. Ethical clearance for this study was granted by the Ethics Committee of Farabi Eye Hospital, with the approval code: IR.TUMS.FARABIH.REC.1400.064. The need for written informed consent was waived by the same Ethic Committee. All methods were performed in accordance with relevant guidelines and regulations.

### Subjects and data acquisition

**Figure 1:**
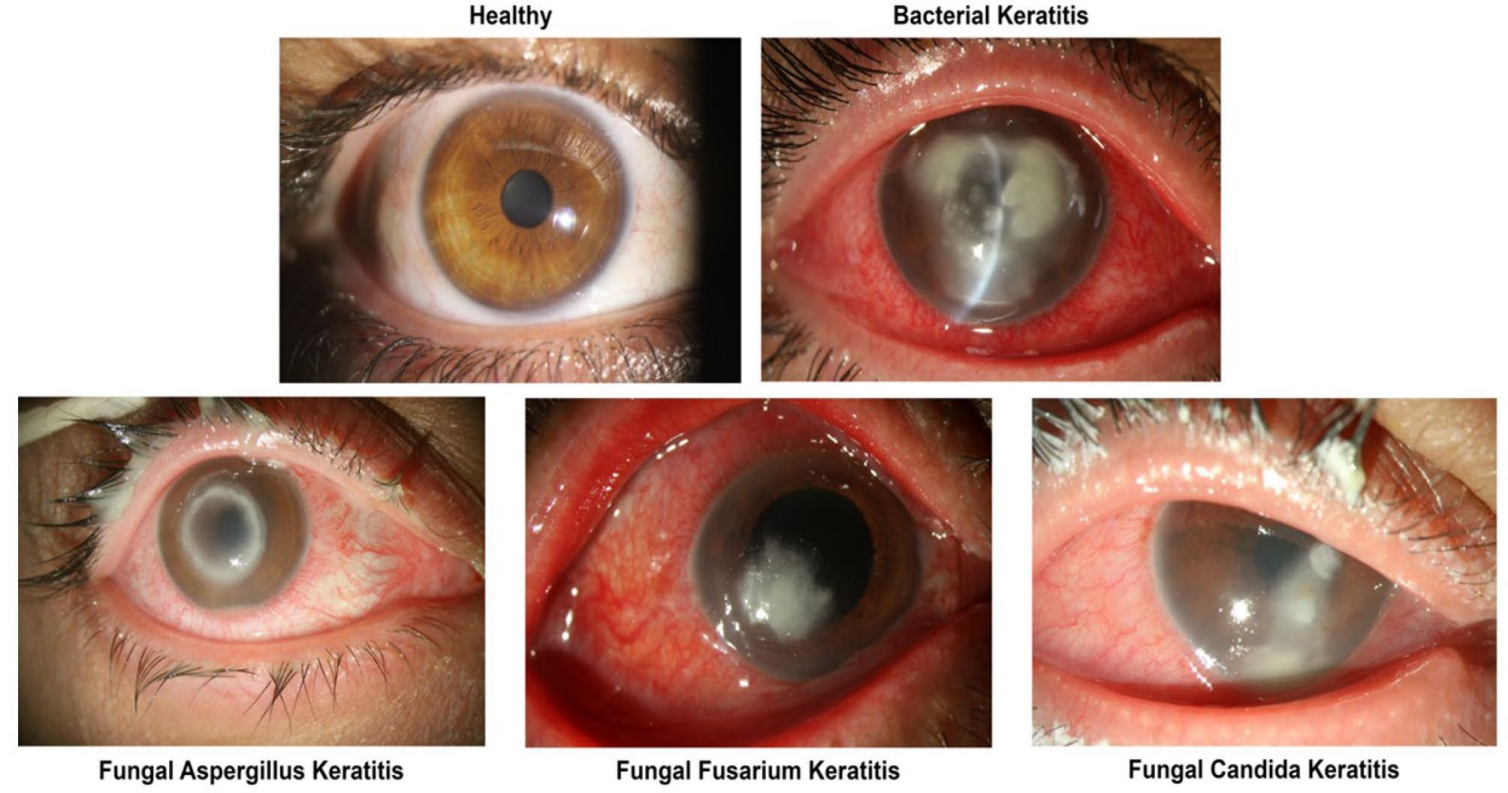
Slit-photo samples of healthy eye and different types of keratitis (e.g., bacterial, *Aspergillus*, *Fusarium*, and *Candida* keratitis) enrolled in the study. Clinical characteristics may vary between different types of keratitis, and different microorganisms may occasionally manifest with a specific infiltrate pattern.

The participants in this study were patients recruited during their visits to the emergency department of Farabi Eye Hospital with a culture-proven diagnosis of bacterial keratitis (BK) or fungal keratitis (FK) between 2014 and 2021. Detailed laboratory investigations are provided here^27^. A population of healthy individuals was enrolled as well. In total, we analyzed data from a collection of 15619 slit-lamp photographs of 1514 participants. In detail, 2505 slit-lamp photographs were taken from 279 healthy individuals, 6761 slit-lamp photographs from 521 patients diagnosed with BK, and 6353 slit-lamp photographs from 714 patients diagnosed with FK. The slit-lamp photographs were captured using a Canon EOS 1300D camera mounted on slit-lamp microscopes, including the Haag-Streit BX900 and Topcon SL-D8 models. Patients with mixed or other types of infection, culture-negative cases, individuals who had previously undergone corneal graft procedures (e.g., penetrating keratoplasty, corneal patch grafts, and amniotic membrane grafts), patients with other significant ocular surface conditions, and those with confounding factors affecting the clinical assessment such as the presence of cyanoacrylate glue patches and bandage contact lens were excluded from the study. Images with poor quality, including those showing extreme gazes or incompletely opened eyelids, were similarly excluded. Finally, the dataset used in this study included 9329 images (from 977 patients); in detail, 2505 images (from 279 patients), 2008 images (from 280 patients), and 4816 images (from 418 patients) of healthy eyes, FK, and BK, respectively. Additionally, images of the FK class have been divided into two subclasses; *Aspergillus/Fusarium* spp. (1643 images from 149 patients) and *Candida* spp. (357 images from 29 patients). Image samples are presented in Fig. 1.

### Dataset and hardware details

The original images had a resolution of 4752 × 3168 pixels and were resized into 300 × 300 due to the limited processing power of the computer used. The configuration of used hardware to debug and run the codes was a laptop with AMD RYZEN 9 6000 SERIES, 32 GB DDR5 RAM and Nvidia GEFORCE RTX 3070 Ti with 8 GB VRAM. All codes were developed using Python 3.10 and deep learning-based codes were simulated using TensorFlow package version 2.12 and Windows Subsystem for Linux (WSL) version 2.0.

Notably, a significant imbalance between the main classes and subclasses was present in the data of our study, which posed a challenge for the training of the models. An approach that is commonly employed to address this issue is the assignment of extra weights, i.e., class weights, to the loss value when the samples of the minority class are misclassified by the model. This method resulted in increased sensitivity for the minority samples and more balanced recognition rates for each class. However, a trial-and-error process is usually required for choosing the appropriate value for class weights. Therefore, three different sets of class weights determined by trial and error were trialed in this study. These values, along with the classes of each model, are presented later.

### Designed models and simulation details

**Figure 2:**
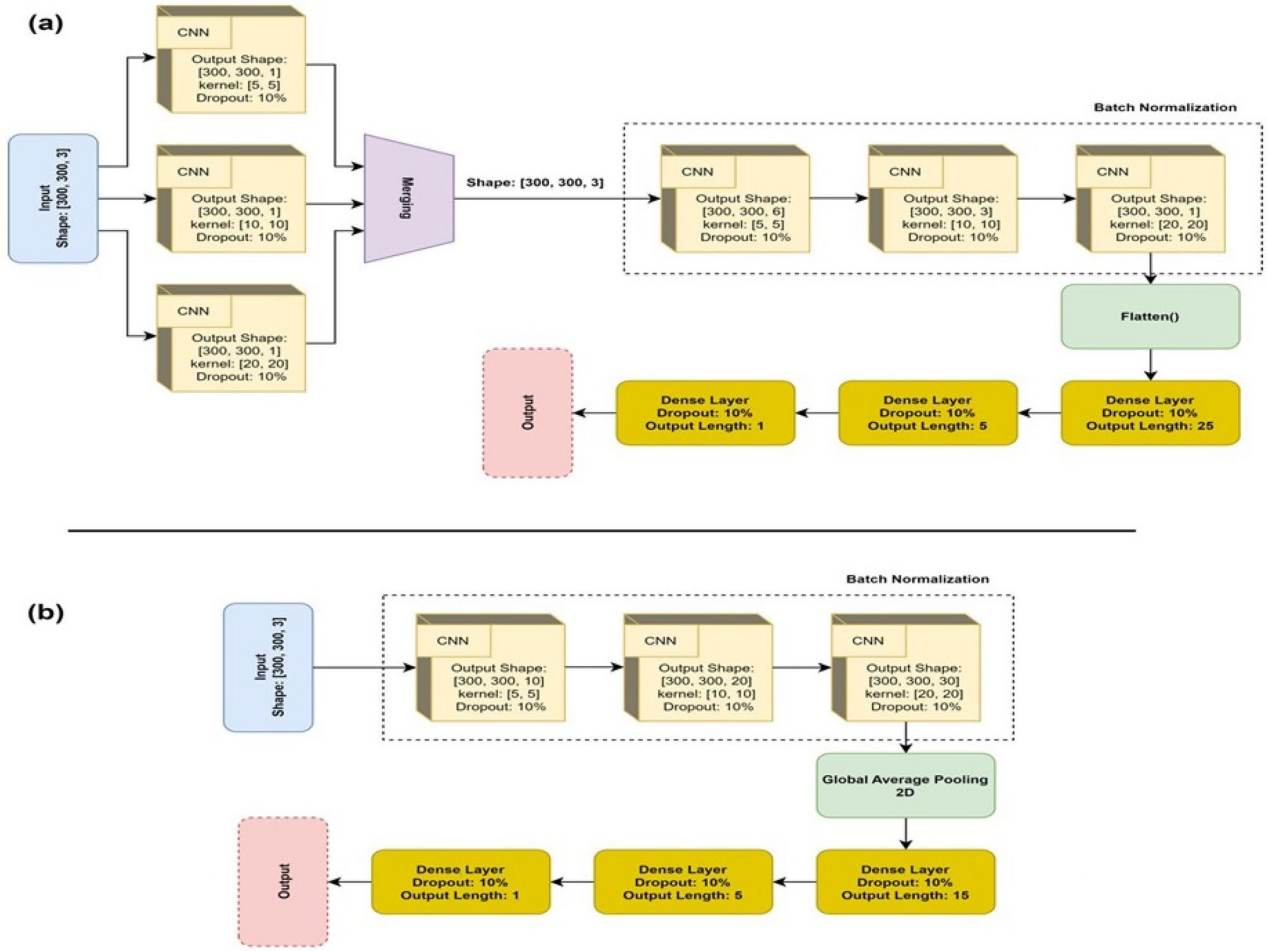
Designation of networks. (**a**) Network 1, Designed to differentiate healthy cases from patients with keratitis, and *Aspergillus* and *Fusarium* from *Candida* in the case of fungal keratitis. (**b**) Network 2, Designed to differentiate bacterial and fungal types of keratitis.

To achieve the goals of our study, two slightly different types of structures were designed to train the models. Both networks were based on Convolutional Neural Networks (CNNs), which are structures that can handle recognition tasks using image-based inputs effectively. These two structures were chosen as the final approaches since they provided results that surpass the other candidates considered for the tasks. Another factor that influenced the selection of the final designs is the minimal size of the chosen networks, which reduces the computational complexity and memory requirements. The details of these two structures are presented in Fig. 2.

As shown in Fig. 2, the main difference between these two networks is the presence of a fusion component in Network 1, which joins three different types of feature maps into one array. This approach fulfills two aims of this study, which are the diagnosis of IK (e.g., differentiation between healthy cases and patients with keratitis) and discrimination of *Aspergillus/Fusarium* spp. from *Candida* spp. in the case of FK. Network 2 is used for fulfilling the other aim of this study, which is to differentiate between bacterial and fungal types of keratitis. In each network, all layers except the last layer use ReLu function for the activation function, while the last layer uses the Sigmoid function.

The data sources, network architectures, and hyperparameters for each model are summarized in Table 1. Although the models use different sizes of datasets for training, validation, and evaluation, they follow the same procedure: 20% of the data is reserved for evaluation using *K*-fold cross-validation with *K* = 5, and the remaining 80% is split into 90% for training and 10% for validation. This means that the training phase uses 72% of the available data, while the validation phase uses 8%. Each model has a unique set of hyperparameters, some of which are shared among all models. As mentioned earlier, although using these values is not mandatory for achieving acceptable results, they are optimized by trial and error based on the limitations of the available hardware.

**Table 1:** Details of each model, including sample size, selected networks, and hyperparameters.

Parameters	Model 1	Model 2	Model 3
Aim of recognition	Healthy vs. keratitis	Fungal vs. bacterial keratitis	*Aspergillus/Fusarium* vs. *Candida* keratitis
Classes			
Class 0 (number of samples)	Healthy (2505)	Fungal keratitis (2008)	*Aspergillus/Fusarium* keratitis (1643)
Class 1 (number of samples)	Keratitis (6824)	Bacterial keratitis (4816)	*Candida* keratitis (357)
Value of *K* (*K*-fold cross-validation)	5	5	5
Training:validation:evaluation ratio (%)	72:8:20	72:8:20	72:8:20
Loss weight of class 0	1	1.5	1
Loss weight of class 1	1	1	25
Batch size	20	15	15
Number of epochs	75	150	250
Learning algorithm	Adam	Adam	Adam
Initial learning rate	0.0001	0.001	0.0001
Loss function	Binary cross entropy	Binary cross entropy	Binary cross entropy

## Results

**Figure 3:**
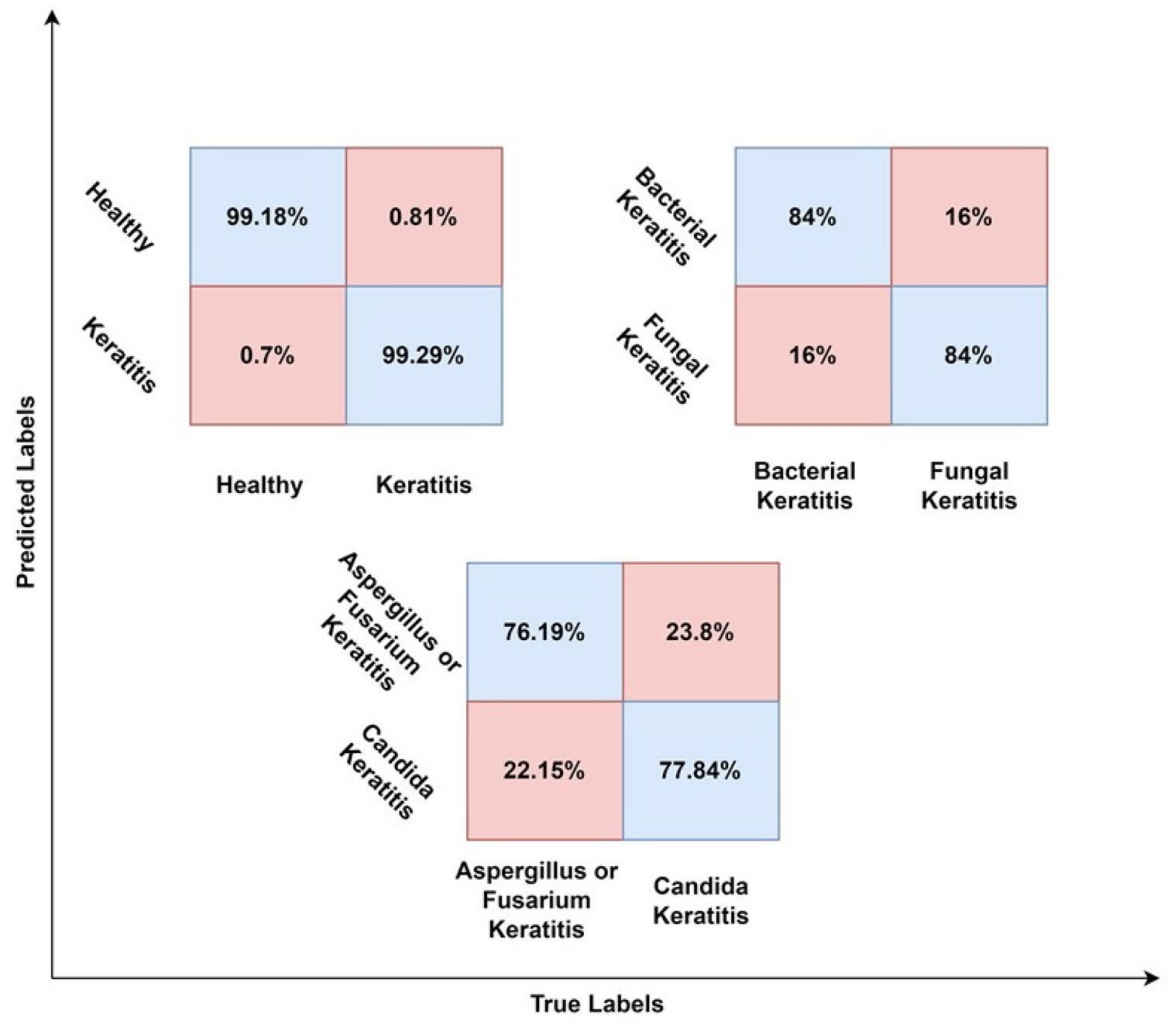
Confusion matrices, regarding each one of three suggested models (e.g., diagnosis of IK (model 1), differentiation between bacterial keratitis and fungal keratitis (model 2), and discrimination between the filamentous type from the yeast type (model 3)).

**Figure 4:**
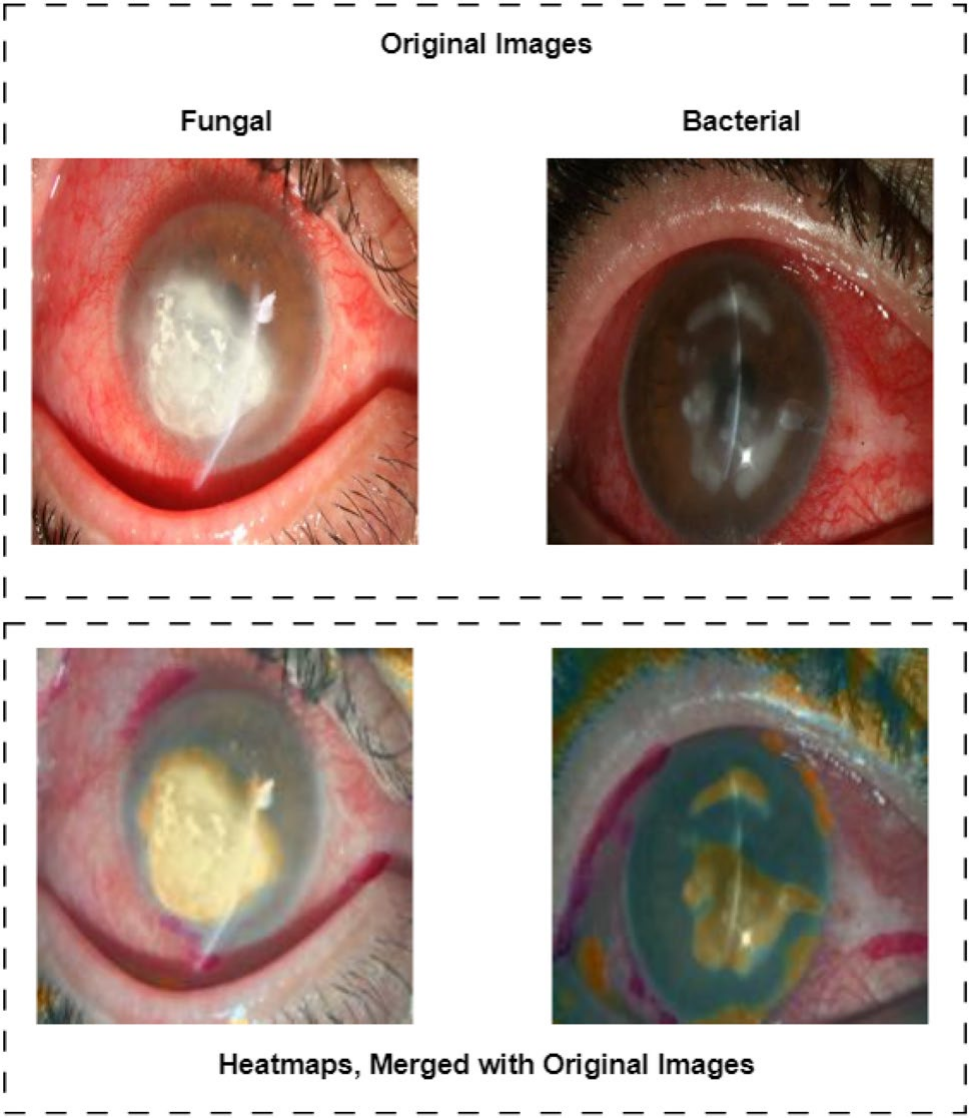
Heatmaps of two sampled images, regarding the fungal and bacterial keratitis, merged with the original inputs.

All results have been validated using the K-fold cross-validation method. The sensitivity and specificity values have been calculated using Eqs. (1) and (2), based on the confusion matrices shown in Fig. 3. Moreover, two heatmap images, extracted from the first layer of the network, designed to differentiate bacterial keratitis from fungal keratitis, are presented in Fig. 4. As it seems, the model is capable of detecting important parts of the input image.

It should be noted that the true positive and true negative values are on the diagonal of the confusion matrix, i.e., the values at (0, 0) and (1, 1) positions, while the false negative and false positive values are off the diagonal, i.e., the values at (1, 0) and (0, 1) positions.

1 $$Sensitivity=\frac{TP}{TP+FN}$$

2 $$Specificity=\frac{TN}{TN+FP}$$

3 $$Accuracy=\frac{TP+TN}{TP+TN+FP+FN}$$

**Figure 5:**
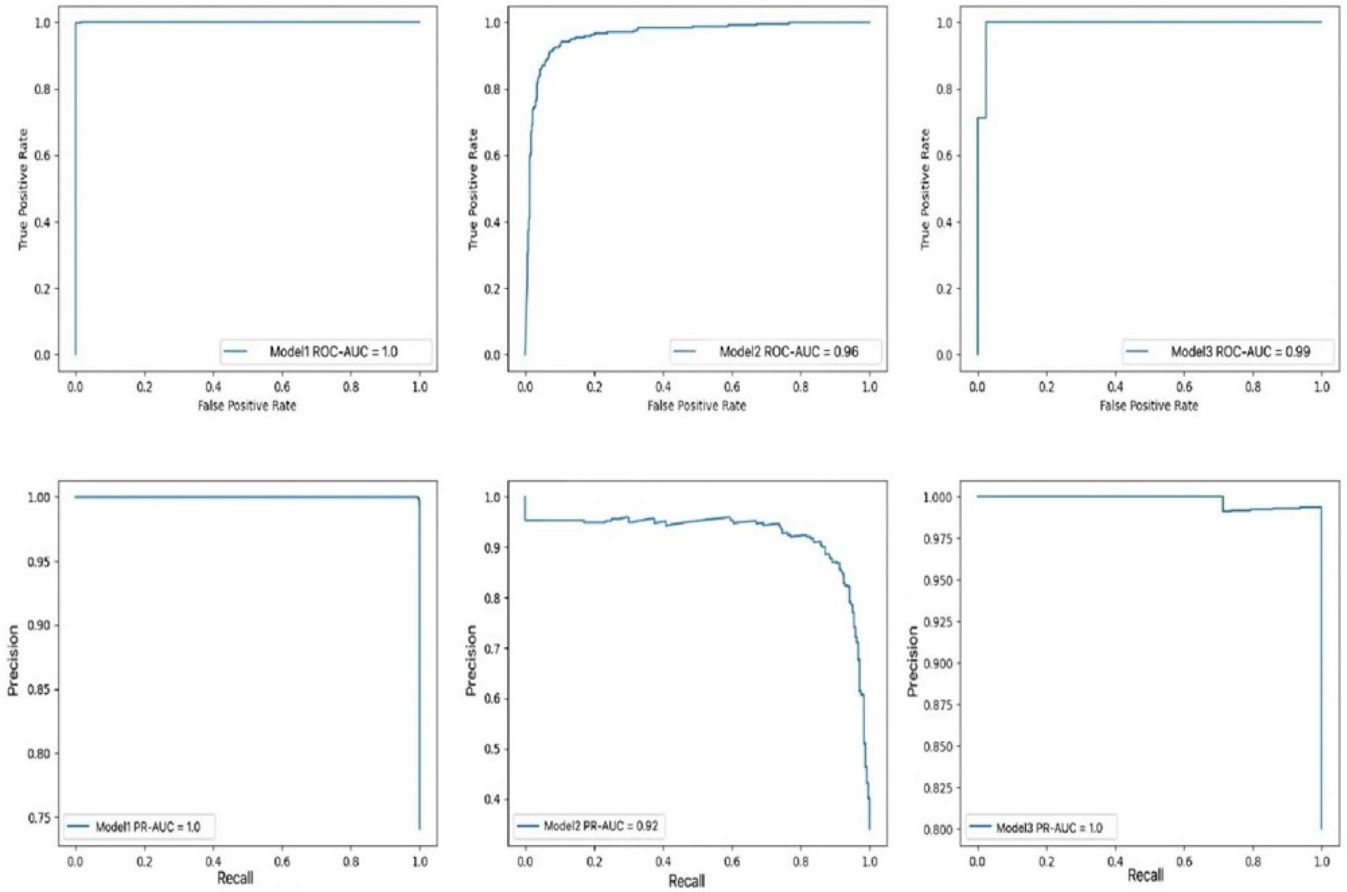
Receiver operating characteristic (ROC) curve and precision-recall curve of the three suggested models.

In addition to the confusion matrices, Table 2 shows the values of other metrics for each model. The results indicate that the accuracy and other metrics decrease as the recognition task becomes more specific. Figure 5 illustrates the ROC curve and precision-recall (PR) curve of the 3 models. The area under the receiver operating characteristic curve (AUC) is mentioned for each model.

**Table 2:** Detailed metrics of our models; sensitivity, specificity, accuracy, receiver operating characteristic-area under the curve, precision recall-area under the curve.

Models	Sensitivity (%)	Specificity (%)	Accuracy (%)	ROC-AUC	PR-AUC
Model 1	99.29	99.19	99.27	0.999	0.999
Model 2	84	84	83.99	0.96	0.92
Model 3	77.47	76.58	77.5	0.99	0.996

## Discussion

Deep Learning represents a subset of machine learning methods that has garnered substantial worldwide attention in recent years. DL employs techniques for learning representations with multiple layers of abstraction, enabling it to handle input data without the necessity for manual feature engineering. It accomplishes this by automatically identifying complex patterns within high-dimensional data by projecting it onto a lower-dimensional space^18,28^. DL utilizes intricate architectures of CNNs. Within these CNNs, software-defined “neurons” work in unison to process data and extracting vital information. These neural networks are meticulously designed to mimic the cognitive processes of the human brain, thus enabling the algorithm to independently evaluate the accuracy of predictions produced during the DL process^29^. Within the field of medicine and healthcare, DL has found multiple applications, particularly in the analysis of medical images. DL systems have demonstrated strong diagnostic capabilities in identifying various medical conditions^18^. Additionally, DL has been leveraged for ophthalmic imaging, specifically with fundus photographs and optical coherence tomography (OCT). Prominent eye diseases where DL techniques have been employed include diabetic retinopathy (DR), glaucoma, age-related macular degeneration (AMD), retinopathy of prematurity (ROP), and corneal ulcers^22,30–32^.

In this study, we developed three CNN-based DL models to diagnose microbial keratitis. Model 1 differentiated normal individuals from patients with microbial keratitis, and obtained accuracy and AUC of 99.3% and 1.0, respectively. Model 2 differentiated FK from BK, and achieved approximate accuracy and AUC of 84% and 0.96, respectively. Model 3 aimed to distinguish between keratitis resulting from two primary subtypes of fungal species: yeasts (*Candida* spp.) and filamentous fungi (*Aspergillus* spp. and *Fusarium* spp.). This model achieved an accuracy of 77.5% and an AUC of 0.99. These three models were designed based on two distinct CNN architectures, as depicted in Fig. 2. Both models were developed and customized by our research team. One notable characteristic of the CNNs is its initial operation on the input data through three parallel layers (Fig. 2a). Each of these layers utilizes a different kernel size. Specifically, the [5, 5] kernel addresses a smaller area of the analyzed image in contrast to the [20, 20] kernel. Models 1 and 3 were constructed with this CNN architecture. In contrast, model 2 demonstrated superior performance when implemented using CNN as illustrated in Fig. 2b. These models demonstrate exceptional performance for different aspects of keratitis. Moreover, given the size of the images used for training and the limitations of the available hardware, it is reasonable to claim that more accurate models can be designed as more detailed data and more powerful computers become available.

In the present study, model 3 was designed to differentiate between keratitis caused by two primary subtypes of fungal species. This particular model attained an accuracy rate of 77.5% along with an AUC of 0.96. This model represents an effort to distinguish between subtypes of fungal keratitis accurately. Its goal is to steer initial empiric treatment towards the most effective and targeted antifungal therapy. The selection of antifungal medications can be influenced by several factors, including their accessibility and the preferences of the treating clinician or infectious disease specialist. In the United States, topical natamycin 5% is both FDA-approved and readily available, and it has demonstrated better outcomes in cases of *Fusarium* keratitis. However, it has poor penetration^10,33^. Topical amphotericin can be the primary choice for treating yeasts and serve as an alternative for filamentous fungi, but it has disadvantages linked to its preparation and stability^33^. To the best of our knowledge, this is the first successful DL-based model in this regard.

Previously, Kuo et al. developed a model to differentiate FK from non-FK with an average accuracy of 69.4%. The AUC in that model was 0.65. Limited number of training data and a high misclassification rate due to the heterogenicity of the non-FK group may have affected the performance of their model^34^. Ghosh and colleagues employed an ensemble of three pre-trained CNNs to differentiate FK from BK, which collectively achieved an accuracy measured by the F1 score (the harmonic mean of precision and recall) of 0.83 and a precision-recall AUC of 0.90^35^. Their findings align closely with the second model in our study and highlights our precision-recall AUC of 0.92. Redd et al., in a multicenter study using ubiquitous hand-held cameras, investigated the diagnostic accuracy of human and AI models. In their study, the AUC of the best CNN architecture, the best human grader, and the ensemble of the best-performing CNN and best-performing human were 0.83, 0.79, and 0.87, respectively. The primary benefit of their MobileNet model lies in its mobility and its potential for use in telemedicine applications^32^. Hung et al. reported an accuracy of about 70% on distinguishing between FK and BK. However, by cropping slit-lamp images using U^2^ segmentation, they attained an accuracy of 80% and an AUC of 0.85^36^. Xu et al. created an advanced deep sequential feature learning model to distinguish between bacterial and fungal keratitis, achieving an accuracy of 84% for fungal keratitis and 65% for bacterial keratitis^37^.

In the case of IK, clinical diagnosis stands as the pivotal initial step for commencing confirmatory tests and delivering efficient empirical treatment to patients, preceding pathogen confirmation^38^. The diagnosis of microbial keratitis is established by considering the patient's medical history and conducting microbiology tests. Although microbiological culture continues to serve as the definitive method for diagnosing IK, our DL-based model, alongside other mentioned templates, has demonstrated the promise of AI in diagnosing IK solely through imaging data. These models facilitate early recognition of potential FK cases and can expedite the start of empirical treatment or facilitate appropriate referral management. These models outperform human experts in some cases. Kuo et al. reported the diagnostic accuracy of non-cornea specialty ophthalmologists and cornea specialists were 67.1% and 75.9%, respectively in differentiating FK from BK. Their DL model reached an accuracy of about 70%, which was higher than non-cornea specialty ophthalmologists^34^. Redd et al. illustrated that even the best human examiners with years of experience (AUC = 0.79) could not outperform their best CNN model (AUC = 0.83)^32^. Xu et al. also reported the average diagnostic accuracy of expert human examiners to be 49.3%, while their DL-based model attained diagnostic accuracies of 53.3% and 83.3% for BK and FK, respectively^37^. In this study, we did not include the diagnostic accuracy of human examiners in distinguishing FK from BK in our dataset. However, considering the comparatively high accuracy achieved by our DL-based model, we anticipate it would surpass the performance of human examiners, but further studies would be required to investigate this claim. It should be noted that in our models, each CNN block represents a convolutional layer. Moreover, it is worth mentioning that in the case of this research, early trials showed that using pretrained networks, such as ResNet, Inception and VGG families, leads to weaker results, compared to training networks from scratch, similar to the approach selected in this study.

There are several limitations to the present study which are important to consider when interpreting the results. To begin with, our study did not control for the conditions and context in which the slit-lamp images were captured. Additionally, the inherent lower image quality due to decreased patient cooperation during severe keratitis cases with heightened symptoms may have impacted our models' performance. Corneal images are known to be more susceptible to artifacts compared to retinal images. The quality of these photographs can be influenced by various factors, including reflections from the slit-lamp light beam, camera flashlight glare, ambient lighting conditions, and overall image brightness. These factors were not controlled in our study. A study by Ghosh et al. demonstrated that the incidence of misclassified data was reduced notably when image brightness was carefully controlled within a specific range^35^. Second, the dataset for this study was collected at a referral medical center, where some patients had already received topical treatments before their examination. Additionally, the ready availability of topical antibacterial medications as over-the-counter options, in contrast to antifungal medications, could have influenced the characteristics of infections. In turn, this disparity may have impacted accuracy of the models as well. Third, the inclusion of multiple images for each eye and the absence of patient matching between the training, validation, and testing groups might have potentially impacted the models’ performance. To mitigate the occurrence of so-called “label leakage”^39^, we implemented a five-fold cross-validation approach, ensuring that both the validation and test datasets remained entirely separate from the training dataset. Fourth, our study exclusively utilized cases with confirmed bacterial and fungal infections through culture testing. Prior confirmations may have led to an artificially inflated diagnostic accuracy for our models. Furthermore, it reduces the practical applicability of these models in a clinical setting. Further studies should investigate the accuracy of our DL models when applied in an office setting before culture positivity is known.

In conclusion, although clinical diagnosis remains a critical initial step in managing infectious keratitis, our DL-based models offer valuable support in the early identification of potential fungal and bacterial keratitis cases and help enable timely treatment or referral management. We have successfully developed three DL-based models tailored for the diagnosis of infectious keratitis, each with its unique role and purpose. These models exhibit high accuracy and hold great promise in enhancing the diagnostic process: Model 1: Geared towards primary healthcare practitioners, this model effectively discerns individuals presenting with ambiguous symptoms of infectious keratitis from otherwise healthy patients. Model 2: Targeted towards expert caregivers and ophthalmologists, this model serves to differentiate fungal keratitis from bacterial keratitis. Its accuracy and performance provide crucial support in ensuring the correct diagnosis and facilitating treatment decisions. Model 3: Designed for the same expert caregivers, this model further distinguishes between the subtypes of fungal species causing keratitis. This precision enables healthcare professionals to guide empiric treatment towards more effective and tailored options, ultimately improving patient outcomes.

## Data Availability

The datasets used and/or analyzed during the current study are available from the corresponding author on reasonable request. Moreover, the codes developed for this study are publicly available in the GitHub repository, addressed: https://github.com/amirrdr/slitlamp-keratitis-1/ (Accessed December 5, 2023).
