# Author Correction: From the diagnosis of infectious keratitis to discriminating fungal subtypes; a deep learning-based study

**DOI:** 10.1038/s41598-025-95224-2

**Published:** 2025-04-24

**Authors:** Mohammad Soleimani, Kosar Esmaili, Amir Rahdar, Mehdi Aminizadeh, Kasra Cheraqpour, Seyed Ali Tabatabaei, Reza Mirshahi, Zahra Bibak-Bejandi, Seyed Farzad Mohammadi, Raghuram Koganti, Siamak Yousefi, Ali R. Djalilian

**Affiliations:** 1 Eye Research Center, Farabi Eye Hospital, Tehran University of Medical Sciences, Tehran, Iran; 2 Department of Ophthalmology and Visual Sciences, University of Illinois at Chicago, Chicago, IL USA; 3 Department of Telecommunication, Faculty of Electrical Engineering, Shahid Beheshti University, Tehran, Iran; 4 Eye Research Center, The Five Senses Health Institute, Rasoul Akram Hospital, Iran University of Medical Sciences, Tehran, Iran; 5 Translational Ophthalmology Center, Farabi Eye Hospital, Tehran University of Medical Sciences, Tehran, Iran; 6 Department of Ophthalmology, University of Tennessee Health Science Center, Memphis, USA; 7 Department of Genetics, Genomics, and Informatics, University of Tennessee Health Science Center, Memphis, USA; 8 Cornea Service, Stem Cell Therapy and Corneal Tissue Engineering Laboratory, Illinois Eye and Ear Infirmary, 1855 W. Taylor Street, M/C 648, Chicago, IL 60612 USA

Correction to: *Scientific Reports* 10.1038/s41598-023-49635-8, published online 14 December 2023

The original version of this Article contained an error in the spelling of the author Zahra Bibak-Bejandi, which was incorrectly given as Zahra Bibak.

The original Article has been corrected.

